# Factors for selective ultrasound screening in newborns with developmental dysplasia of the hip **(**DDH**)**

**DOI:** 10.3389/fsurg.2022.1038066

**Published:** 2022-10-24

**Authors:** Chanika Angsanuntsukh, Tanyaporn Patathong, Krongkaew Klaewkasikum, Witoon Jungtheerapanich, Tanyawat Saisongcroh, Pornchai Mulpruek, Patarawan Woratanarat

**Affiliations:** ^1^ Department of Orthopaedics, Faculty of Medicine Ramathibodi Hospital, Mahidol University, Bangkok, Thailand; ^2^ Orthopaedic Unit, Weingsra Crown Prince Hospital, Surat Thani, Thailand

**Keywords:** Barlow test, breech presentation, hip abduction, Ortolani test, risk

## Abstract

**Background:**

Hip ultrasound screening for DDH provides better sensitivity compared to physical examination. Due to a lower prevalence and limited resources, selective hip ultrasound in newborns at risk could be considered a proper screening protocol in Thailand and Asian countries.

**Objective:**

This study was aimed to evaluate risk factors and define criteria for selective screening.

**Methods:**

A case-control study was conducted in 2020. All newborns with hip ultrasound screening were included. Cases were defined as newborns with abnormal hip ultrasounds, while controls were those with normal studies. Inter and intra-rater reliability were evaluated. All factors were analyzed using univariate and multivariate logistic regression. The model performance was tested by Hosmer-Lemeshow goodness of fit. Internal validity was performed by the split data method. Area under the receiver operating characteristic (ROC) curve was estimated.

**Results:**

Ninety-five newborns (29 cases and 66 controls) were included. Eighty percent of cases and 58% of controls were female. The gestational age was 36.6 and 37.7 weeks in case and control, respectively. Female, breech presentation, positive Ortolani test, positive Barlow test, and limited hip abduction were significant factors with odds ratio of 2.82, 5.12, 34.21, 69.64, and 5.48, respectively. The final model included breech presentation, positive Ortolani test, and positive Barlow test. The model cut-off value 15.02 provided sensitivity (93.10%) and specificity were (80.30%). The area under the ROC curve was 0.9308. The split data remained significant internal validity for all factors with *p*-value < 0.05.

**Conclusion:**

Careful history taking and physical examination are essential to identify the risk factors for DDH. Newborns with breech presentation, positive Ortolani test and positive Barlow test should be screened by hip ultrasound.

## Introduction

Developmental dysplasia of the hip (DDH) is a disorder of abnormal development between the head of the femur and acetabulum, which can result in dysplasia, subluxation, and dislocation (1, 2). Clinical examination is used for DDH screening in many countries; however, this method has had low sensitivity of 28.1% and a specificity of 94.5% (3). Ultrasound screening has a sensitivity of 88.5% and specificity of 96.7% (4), which gained acceptance as the most effective method for early diagnosis of DDH (5–8). Therefore, hip ultrasonography is being used as a universal ultrasound in Europe to screen newborns. Whereas, the USA implements selective ultrasound screening for newborns who has risk factors of DDH i.e., breech and family history of DDH (2, 5, 9).

There is a significant variability in incidence within each racial group by geographic location; the incidence of DDH in the Caucasian population was higher than Asian population (10). In Thailand, the incidence per 1,000 live birth of DDH is 0.5 (10), for which, clinical examination was used as the primary technique for DDH screening. DDH screening is based on physical examination by general practitioners. While hip ultrasonography could not be done in all newborns due to resource scarcity, high cost, and increased workload for limited pediatric orthopaedists, it has resulted in delayed diagnosis of DDH. Late presentation of DDH with hip dislocation in walking age is still a problem. Late diagnosis of DDH is associated with a significant risk of poorer outcomes, including increased likelihood of surgery, more invasive surgical procedures, more extended hospital stays, and early osteoarthritis of the hip, as well as increased healthcare costs (11–13). Therefore, an early diagnosis is essential for an early treatment to reduce the possibility of hip osteoarthritis in young adults (14).

This study aimed to find the risk factors of DDH to create a model to selectively screen Thai newborns. This study will benefit by early diagnosis of DDH and treatment, optimization of the ultrasound screening tool, and reduction of unnecessary costs.

## Methods

### Study design and setting

We conducted a case-control design to examine the association between various risk factors and DDH in newborns and design clinical criteria for ultrasound screening in Thai newborns. Cases were selected from Ramathibodi hospital's database of Orthopaedics department between 2009 and 2020. Controls were selected from Ramathibodi hospital's database of the Department of Pediatrics between 2009 and 2020. Institutional Review Board (IRB) of faculty of Medicine, Ramathibodi Hospital, Mahidol University approved the study with reference number COA. MURA2021/273.

### Participant

#### Case definition, inclusion,and exclusion

A case was defined as a newborn of any gender diagnosed with DDH by ultrasound techniques in any position of the standard Harcke's techniques (15–17) (within 2 weeks after birth) in Ramathibodi Hospital between 2009 and 2020. We set the criteria to include newborns with hip ultrasound screening within 2 weeks after birth because newborn with hip laxity or mild instability could be spontaneously resolved within 2–3 weeks.

#### Control definition, inclusion and exclusion

A control was defined as a newborn who was born at our institute between 2009 and 2020 with normal hip ultrasound in all positions of the standard Harcke's technique (15–17) within 2 weeks after birth. Controls were matched with cases for age and nationality.

### Hip ultrasound and reliability

The definitive diagnosis of DDH or not DDH was made based on hip ultrasound. Morphology, stability, and laxity was assessed. The ultrasonographic interpretation were normal (stable and normal in all aspects), dysplasia (abnormal morphology with acetabular coverage <50%) (18) laxity (hip incongruency in adduction), subluxable (subluxation in Barlow test), dislocatable (dislocation in Barlow test) and dislocated hip. All the newborns with abnormal hip ultrasound were diagnosed as positive cases for DDH. We assessed the reliability of the ultrasound interpreters in 10 consecutive newborns, using convenient sampling. The inter and intra-rater reliability of hip assessment and ultrasound were performed by two experienced pediatric orthopaedics surgeons (trained for hip ultrasound from Nemours/Alfred I. duPont Hospital for Children, Wilmington, Delaware) using the standard Harcke's techniques (15–17).

### Data collection

The baseline characteristics and clinical assessments were obtained from hospital databases. We used the DDH risk factors based on the meta-analysis (19) and previous studies (5, 20–23), such as gestational age, gender, breech presentation, family history, firstborn child, associated anomalies, Ortolani test (5), Barlow test (5), limited hip abduction, Galeazzi sign (23). The outcome was the ultrasound result in any position of the standard Harcke's methods (15–17).

### Statistical analysis

The difference between the case and control groups were assessed using t-test and chi-square test. Univariate logistic regression was used to evaluate each risk factor independently – and, variables with *p*-value <0.05 were included in a multivariate logistic regression model. The multivariate analysis used the forward stepwise method to assess the risk factor. The model performance was tested by Hosmer-Lemeshow goodness of fit, and area under the receiver operating characteristic (ROC) curve. Odds ratio (OR) was used to measure the association. Odds ratios of significant risk factors from the final model was enumerated and summed scores. The model classification (high vs. low risk) was set as at the optimal cutoff value based on the best sensitivity and specificity. Internal validity was assessed using the split data method. All analyses were performed using the STATA software package, version 16.0 (Stata Corp, College Station, Texas, USA). A two-sided *p*-value of less than 0.05 was considered as the threshold for statistical significance.

## Results

Ninety-five newborns (29 cases and 66 controls) were included. Eighty percent of cases and 58% of controls were female. The gestational age was 36.6 and 37.7 weeks in case and control, respectively.

Significant risk factors of DDH included breech presentation (*p*-value = 0.002), positive Ortolani test (*p*-value < 0.001), positive Barlow test (*p*-value < 0.001) and limited hip abduction (*p*-value = 0.022). No difference was observed for gestational age, sex, family history of DDH, firstborn child, associated anomaly, and positive Galeazzi sign (Table 1). Characteristic of cases was in (Table 2). Fifty percent of them had hip subluxation followed by hip dysplasia, laxity and dislocation.

**Table 1:** Baseline characteristic.

Variable	Cases (*n* = 29)	Controls (*n* = 66)	*p*- value
Gestational ages (mean, SD)	36.6 (7.27)	37.7 (4.84)	0.378
Female gender (*n*, %)	23 (79.31)	38 (57.58)	0.062
Breech presentation (*n*, %)	12 (41.38)	8 (12.12)	0.002^a^
Family history of DDH in 1st degrees relatives (*n*, %)	0 (0)	1 (1.52)	1.000
First born child (*n*, %)	19 (65.52)	39 (59.09)	0.650
Associated anomalies (*n*, %)	4 (13.79)	3 (4.55)	0.195
Positive Ortolani test (*n*, %)	10 (34.48)	1 (1.52)	<0.001^a^
Positive Barlow test (*n*, %)	15 (51.72)	1 (1.52)	<0.001^a^
Limited hip abduction (*n*, %)	6 (20.69)	3 (4.55)	0.022^a^
Positive Galeazzi sign (*n*, %)	2 (6.90)	2 (3.03)	0.583

^a^ Significant (*p* < 0.05), SD = standard deviation, CI = Confidence interval.

**Table 2:** Characteristic of cases with abnormal hip ultrasound.

**Characteristic** (%)	**N = 29 cases**
Female	23 (79.31)
Right Side	22 (75.86)
Bilateral	13 (44.83)
**Result of Hip Ultrasound**	**N = 42 hips**
Laxity	7 (16.67)
Dysplasia	8 (19.05)
Subluxation	21 (50)
Dislocation	6 (14.28)

The quality of ultrasonographic assessment: two pediatric orthopedic surgeons independently did the ultrasound in 10 newborns. The agreement of ultrasound results was 96.3%.

Univariate analysis showed a significant difference of DDH between two groups in five predictors including female gender (*p*-value = 0.047), breech presentation (*p*-value = 0.002), positive Ortolani test (*p*-value < 0.001), positive Barlow, test (*p*-value < 0.001), and limited hip (*p*-value = 0.023). Female newborns who had breech presentation, positive Ortolani test, positive Barlow, test, and limited hip were more likely to have DDH (OR [95%CI] = 2.82 [1.02, 7.85], OR [95%CI] = 5.12 [1.79, 14.55], OR [95%CI] = 34.21 [4.11, 284.51], OR [95%CI] = 69.64 [8.49, 571.57], OR [95%CI] = 5.48 [1.26, 23.73], respectively) (Table 3).

**Table 3:** Univariate logistic analysis and odd ratio.

Variables	Odds ratio	95% CI	*p*-value
Gestational ages (<37 weeks)	0.97	0.90, 1.04	0.397
Female gender	2.82	1.02, 7.85	0.047^a^
Breech presentation	5.12	1.79, 14.55	0.002^a^
Family history of DDH in 1st degrees relatives	1	–	–
First born child	1.32	0.53, 3.26	0.555
Associated anomalies	3.35	0.70, 16.10	0.130
Positive Ortolani test	34.21	4.11, 284.51	0.001^a^
Positive Barlow test	69.64	8.49, 571.57	<0.001^a^
Limited hip abduction	5.48	1.26, 23.73	0.023^a^
Positive Galeazzi sign	2.37	0.32, 17.71	0.400

^a^ Significant (*p* < 0.05), CI = Confidence interval.

**Figure 1:**
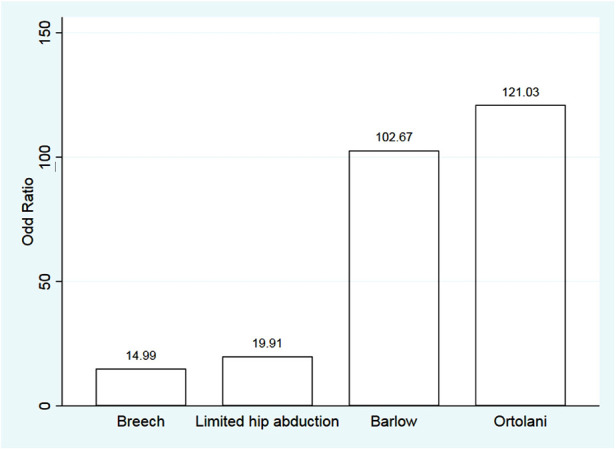
Odds ratio of 4 remained variables.

Of the initial 10 possible predictors, 5 that remained with high odds ratios were female gender, breech presentation, positive Ortolani test, positive Barlow test, and limited hip abduction. Multivariate analysis showed a significant association with breech presentation, positive Ortolani test, positive Barlow test, and limited hip abduction (*p*-value = 0.005, <0.001, <0.001, and 0.009, respectively) (Table 4 and Figure 1).

**Table 4:** Multivariate logistic regression analysis of 4 remaining variables.

Variable	Adjusted odds ratio	95% CI	*p*-value
Breech presentation	14.99	2.47, 90.94	0.003
Positive Ortolani test	121.03	9.36, 1565.35	<0.001^a^
Positive Barlow test	102.67	8.43, 1250.21	<0.001^a^
Limited hip abduction	19.91	2.14, 184.97	0.009

^a^ Significant (*p* < 0.05), CI = Confidence interval.

**Figure 2:**
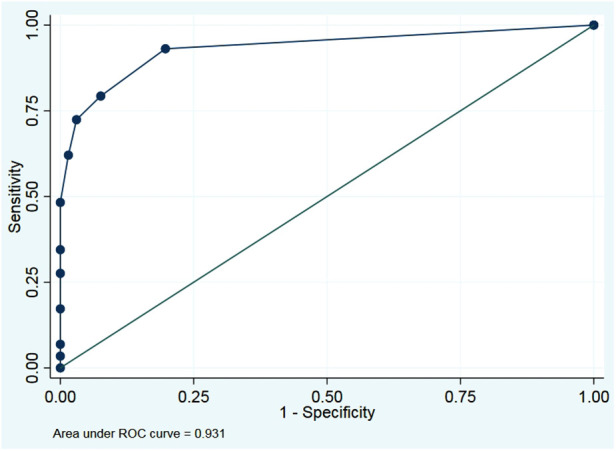
Area under the receiver operating characteristic (ROC) curve at cutoff value 15.02.

The final model included breech presentation, positive Ortolani test, positive Barlow test, and limited hip abduction. The best model cut-off value was 15.02. Sensitivity and specificity were 93.10% and 80.30%, respectively. The area under the ROC curve was 0.931 (Tables 5, 6 and Figure 2). Internal validation was done to estimate the potential for optimism and overfitting in model performance. We randomly split data into two sets with a ratio (70:30) for a development sample and a validation sample. The result of the four predictors remained significant (Table 7).

**Table 5:** Cutoff values and diagnostic ability for DDH.

Score	Sensitivity (%)	Specificity (%)	LR+	LR−
≥0.346	100.0	0	1.00	–
≥15.02	93.10	80.30	4.73	0.09
≥19.94	79.31	92.42	10.47	0.22
≥102.7	72.41	96.97	23.89	0.28
≥117.6	62.07	98.48	40.97	0.38
≥121.0	48.28	98.48	31.86	0.52

LR+ = likelihood ratio of positive test, LR− = likelihood ratio of negative test.

**Table 6:** The scoring system.

Breech presentation (14.99)	Ortolani test (121.03)	Barlow test (102.67)	Limited hip abduction (19.91)	Score
No	No	No	No	0
Yes	No	No	No	14.99
Yes	No	No	Yes	34.9
Yes	No	Yes	No	117.66
Yes	No	Yes	Yes	137.57
Yes	Yes	No	No	136.12
Yes	Yes	Yes	No	28.69
Yes	Yes	No	Yes	155.93
Yes	Yes	Yes	Yes	258.87
No	Yes	No	No	121.03
No	Yes	Yes	No	223.7
No	Yes	No	Yes	140.94
No	Yes	Yes	Yes	243.61
No	No	Yes	No	102.67
No	No	No	Yes	19.91
No	No	Yes	Yes	122.58

**Table 7:** Internal validity.

Variable	Coef.	95% CI	*p*-value
Breech presentation	2.85	0.96, 4.74	0.003^a^
Positive Ortolani test	3.83	1.15, 6.51	0.005^a^
Positive Barlow test	3.38	0.71, 6.05	0.013^a^
Limited hip abduction	3.79	1.08, 6.51	0.006^a^

^a^ Significant (*p* < 0.05), Coef = Coefficient, CI = Confidence interval.

## Discussion

Delayed diagnosis of DDH related to a significant morbidity and complexity of the treatments. Screening of the DDH in newborns is important to obtain the early diagnosis. Hip ultrasound screening for DDH provides better sensitivity compared to physical examination. Due to a lower prevalence and limited resources, selective hip ultrasound in newborns at risk could be considered as a proper screening protocol in Asian countries, including Thailand.

There is an unclear conclusion about the risk factors to identify newborns at risk for DDH (20, 21). Moreover, the screening criteria may be different in each country. From previous studies, the risk factors are prematurity, oligohydramios, positive family history, breech presentation, postnatal traditional swaddling, female, clicking hip, and abnormal physical examination (6). While in this study, the criteria to warrant selective ultrasound included breech presentation, positive Ortolani test, positive Barlow test, and limited hip abduction. Other factors were found insignificant. This might be caused by the incidence of DDH in our study was very low and the other risk factors were rarely positive resulting in inadequate sample size to detect level of significance.

Abnormal physical examination of hip has been found to be a strong factor related to DDH (19, 21), which was similar to our results. Roposch A et al. (21) reported an odds ratio of abnormal physical examination (OR 53.91, 95% CI 31.35, 92.71), but did not specify the physical examination test as they aimed to create a risk prediction tool for maternity ward doctors. Meta-analysis of de Hundt M et al. (19) reported odds ratio of abnormal physical examination of clicking hip (OR 8.6, 95% CI 4.5, 16.6); however, they did not clearly state the criteria of clicking hip. Our study reported odds ratios of each abnormal physical examination including positive Ortolani test (OR 121.03, 95%CI 9.36, 1565.35; *p* < 0.001), positive Barlow test (OR 102.67, 95%CI 8.43, 1250.21; *p* < 0.001), and limited hip abduction (OR 19.91, 95%CI 2.14, 184.97; *p* = 0.009). The ORs of breech presentation in the European population were ranged between (OR 1.90–5.7) (5, 19, 20). Our study found more chance of DDH in Asian population who have a breech presentation (OR 14.99, 95%CI 2.47, 90.94; *p* = 0.003). The explanation might be the proportion of breech presentation in our study was very high (12 out of 29 cases; 41.38%) compared to controls (8 out of 66 newborns, 12.12%), Table 1. Moreover, adjusted by other strong risk factors may lead to large magnitude of association (Table 4).

Female gender was found as a risk factor for DDH (OR 2.27–5.8) (5, 19, 21) which was similar to our results from univariate analysis (OR 2.82, 95%CI 1.02, 7.85; *p* = 0.047); however, female gender was not significant in the multivariate analysis. In our analysis, family history of DDH was not significantly associated with DDH, which was the difference from previous studies (19–22). The contrast of these results might be because our study had a smaller sample size compared to earlier studies in the European population. DDH is not a common disease in Thailand, and the incidence of DDH in Asian countries is lower than in Europe (10), for which some factors might need a larger sample size.

We did an internal validation by randomly splitting data to correct predictive performance measures for optimism, of which four predictors gave significant results (*p* < 0.050). Our final model demonstrated excellent discrimination (area under ROC = 0.931) with four potential factors.

The strength of our study is that we developed the model with a high degree of precision. Our study used widely-accepted perinatal risk factors of DDH based from meta-analysis (19) and previous studies (5, 19–22). Two well-trained pediatric orthopaedic doctors measured the outcome with an excellent agreement in ultrasound results (96.3%). The limitations of our study include small number of patients, which might have affected the insignificant association of some risk factors such as female and family history of DDH, and imprecise confidence interval. There were 2 cases diagnosed DDH and did not have any risk factors (breech presentation, positive Ortolani test, positive Barlow test, limited hip abduction). One case had right hip subluxation and was treated by Pavlik harness. The other one was female with hip dysplasia. Both of them had neither history of oligohydramnios, family DDH, nor the first born child. It implies that suggested risk factors could miss 2 out of 29 DDH cases (6.9%). While 66 out of 95 newborns (69.5%) avoided unnecessary ultrasound screening. Further newborn recruitment may detect more significant risk factors, and perfectly capture all DDH.

In Thailand, orthopaedic surgeons are not the primary physicians who perform physical examination for detecting DDH. DDH screening is based on physical examination experiences by general practitioners or pediatricians. Only abnormal physical examination cases were referred to specialized hospitals. Although, universal ultrasound cannot be applied for all Thai newborns due to the limited resource and cost. Our model can be used for selective ultrasound screening to prevent the delayed diagnosis of DDH in an individual at-risk newborn. The model includes the clinical variables which used widely-accepted perinatal risk factors of DDH which in-significant factors should be noted as a precaution. However, it is critical to select all newborns having DDH and be treated adequately. We recommended that the high risk patients should be screen by ultrasound, while the patient with some risk factor, for example female, or family history of DDH, should have adequate follow up and repeat physical examination. Moreover, future studies suggest increasing the sample size to see the robustness of the result.

## Conclusion

Careful history taking and physical examination are essential to identify the risk factors for DDH. Newborns with breech presentation, positive Ortolani test and positive Barlow test should be screened by hip ultrasound.

## Data Availability

The original contributions presented in the study are included in the article/Supplementary Material, further inquiries can be directed to the corresponding author/s.
